# Development and Characterization of Electrospun Poly(3-hydroxybutyrate-co-3-hydroxyvalerate) Biopapers Containing Cerium Oxide Nanoparticles for Active Food Packaging Applications

**DOI:** 10.3390/nano13050823

**Published:** 2023-02-23

**Authors:** Kelly J. Figueroa-Lopez, Cristina Prieto, Maria Pardo-Figuerez, Luis Cabedo, Jose M. Lagaron

**Affiliations:** 1 Novel Materials and Nanotechnology Group, Institute of Agrochemistry and Food Technology (IATA), Spanish Council for Scientific Research (CSIC), Calle Catedrático Agustín Escardino Benlloch 7, 46980 Paterna, Spain; 2 Polymers and Advanced Materials Group (PIMA), Universitat Jaume I (UJI), Avenida de Vicent Sos Baynat s/n, 12071 Castellón, Spain

**Keywords:** PHBV, cerium oxide, oxygen scavengers, electrospinning, antioxidant activity, antimicrobial activity

## Abstract

Food quality is mainly affected by oxygen through oxidative reactions and the proliferation of microorganisms, generating changes in its taste, odor, and color. The work presented here describes the generation and further characterization of films with active oxygen scavenging properties made of poly(3-hydroxybutyrate-co-3-hydroxyvalerate) (PHBV) loaded with cerium oxide nanoparticles (CeO_2_NPs) obtained by electrospinning coupled to a subsequent annealing process, which could be used as coating or interlayer in a multilayer concept for food packaging applications. The aim of this work is to explore the capacities of these novel biopolymeric composites in terms of O_2_ scavenging capacity, as well as antioxidant, antimicrobial, barrier, thermal, and mechanical properties. To obtain such biopapers, different ratios of CeO_2_NPs were incorporated into a PHBV solution with hexadecyltrimethylammonium bromide (CTAB) as a surfactant. The produced films were analyzed in terms of antioxidant, thermal, antioxidant, antimicrobial, optical, morphological and barrier properties, and oxygen scavenging activity. According to the results, the nanofiller showed some reduction of the thermal stability of the biopolyester but exhibited antimicrobial and antioxidant properties. In terms of passive barrier properties, the CeO_2_NPs decreased the permeability to water vapor but increased the limonene and oxygen permeability of the biopolymer matrix slightly. Nevertheless, the oxygen scavenging activity of the nanocomposites showed significant results and improved further by incorporating the surfactant CTAB. The PHBV nanocomposite biopapers developed in this study appear as very interesting constituents for the potential design of new active organic recyclable packaging materials.

## 1. Introduction

Food products are subjected to different conditions (temperature, water vapor, oxygen, ultraviolet light, microorganisms, mechanical stress, etc.) throughout the supply chain, which could alter food quality. In this sense, conventional food packaging technology was originally developed to contain and protect food products from these external factors [1]. Nevertheless, recently food packaging has evolved, incorporating novel active, passive, and intelligent constituents to prolong expiration dates and to preserve or increase quality, safety, and integrity [2,3]. In addition, taking into account the current environmental concern, there is an increased attention in renewable biopolymer-based packaging materials as eco-friendly and sustainable alternatives to substitute traditional non-biodegradable petroleum-based plastics [4,5].

Polyhydroxyalkanoates have increasingly become an attractive alternative to non-renewable polymers for food packaging applications following the circular economy (PHAs) principles [6,7] due to their biocompatibility and wide range of physical properties [8]. PHAs can be defined as homo-, co-, and terpolymers [9] and classified in relation to the number of carbon atoms in the monomer into: short-chain-length PHAs (scl-PHAs) with 3 to 5 carbons, medium-chain-length PHAs (mcl-PHAs) with 6 to 14 carbons, and long-chain-length PHAs (lcl-PHAs) with more than 14 carbons [10,11]. Among them, the copolymer PHBV has a strong potential for food packaging applications since it has considerably lower crystallinity and melting temperature (T_m_), which diminish as the percentage of HV fraction in the polymer increases [12], exhibits enhanced flexibility, ductility, and elongation at break, and augmented tensile strength as a consequence of a reduction in the Young’s modulus as the fraction of HV increases [13].

The novel generation of packaging has an active part in the conservation of food quality throughout the supply chain. According to Regulation (CE) No. 450/2009 (29/05/2009), active packaging consists of a material that incorporates intentionally active compounds that release and absorb substances into or from either the environment or the packaged food [14]. Active compounds in active packaging are specially catalogued as either scavengers, which can remove unwanted substances from the food environment, or active-releasing (emitters), which provide substances to packaged food or into headspace, conferring long-term antioxidant and/or antimicrobial properties [15]. Thus, oxygen scavengers have the ability to capture the oxygen present inside the packaging material, creating an oxygen-free atmosphere, which can reduce the oxidative, enzymatic, and microbial reactions in food [16]. Oxygen scavengers are normally iron-based, in which the mechanism of action is principally due to the oxidation of iron when moisture is present. Thus, ferrous oxide (Fe^2+^) is transformed to ferric oxide (Fe^3+^). Another widely known oxygen scavenger is palladium, which reacts based on the adsorption of both hydrogen and oxygen on palladium surface (by chemisorption), followed by a chemical reaction at the surface and desorption of water into gas phase [17,18]. These materials are classified as moisture-activated oxygen scavengers, and consequently they highly depend on the relative humidity of the environment and are capable of reducing oxygen concentration within the food package to percentages lower than 0.01% [19].

An alternative to common oxygen scavengers is cerium oxide (CeO_2_), which has attracted much attention due to its capability to transition from Ce^3+^ to Ce^4+^ states reversibly, which is responsible for the catalytic, antioxidant, anti-inflammatory, and anti-bacterial activity characteristics of these nanoparticles [20]. CeO_2_NPs has numerous applications that include catalysis, biomedicine, energy, electrochemistry, photoelectronics, novel materials with enhanced antimicrobial, superhydrobophic, anticorrosive or mechanical properties or sensors, among others due to their special structure and atomic characteristics in comparison to other systems [21,22,23,24,25,26,27,28,29,30,31].

One of the most promising methods to incorporate these nanoparticles into biopolymeric matrixes is electrospinning [23]. This simple, cost-effective, and versatile technique generates ultrathin polymeric fibers with control of fiber diameters and porosity [24]. This technique offers advantages; the process is carried out at room temperature and provides optimum encapsulation efficiency, which maintains the bioactivity of the encapsulated substances and offers their sustained and controlled release. These advantages have permitted the development of active and high-barrier food packaging by means of electrospinning [32]. In this sense, this technology has proven very valuable for the production of active packaging based on poly(3-hydroxybutyrate) (PHB) and polycaprolactone (PCL) loaded with palladium nanoparticles (PdNPs) for the development of monolayer and multilayer materials with oxygen scavenging capacity [33,34].

In this context, this research aimed at assessing the capacity of the electrospinning technology to produce active and passive barrier biopaper films from annealed electrospun PHBV comprising CeO_2_NPs and CTAB as a surfactant. This film could be potentially of use as an interlayer or coating in a multilayer concept for food packaging applications. Firstly, the electrospun PHBV fibers and resultant biopapers after annealing were characterized based on their optical and thermal properties, morphology, crystallinity, and oxygen scavenging capacity. Secondly, the films with the best oxygen scavenging performance were chosen and characterized according to their mechanical, barrier, antimicrobial, and antioxidant properties.

## 2. Materials and Methods

### 2.1. Materials

PHBV (ENMAT^TM^ Y1000P) was purchased from Tianan Biologic Materials (Ningbo, China) and provided in the form of pellets by NaturePlast (Ifs, France). As stated by the producer, the material is characterized by a density of 1.23 g/cm³, a melt flow index (MFI) of 5–10 g/10 min (190 °C, 2.16 kg), and a 3HV fraction of 2 mol.-%. The cerium (IV) oxide (CeO_2_) nanoparticles, with particle size < 25 nm calculated by means of the Brunauer, Emmett, and Teller (BET) method, were provided by Sigma Aldrich (Madrid, Spain). According to the literature, the cytotoxicity (LC50) of CeO_2_ was 1000 µg/mL measured in MCF7 cells [35], and the oral acute toxicity was (LD50) >5000 mg/kg measured in rats [36]. Hexadecyltrimethylammonium bromide (CTAB) (99%); 2,2,2-trifluoroethanol (TFE) (≥99%); and D-limonene (98%) were provided by Sigma Aldrich (Madrid, Spain).

### 2.2. Electrospinning Process

First, PHBV was mixed with the solvent TFE at a concentration of 10 wt.%. Nanoparticles of CeO_2_ were added (0.5, 1.0, 1.5, 2.0, 5.0, and 10 wt.%) in relation to the PHBV. Hexadecyltrimethylammonium bromide (CTAB) is a cationic surfactant, not yet approved for use in foods but acceptable for pharmaceutical and food contact applications [37], which has been studied for the development of novel materials for food packaging applications [38,39,40]. It was added at a concentration of 0.5 wt.% to the PHBV mixture to enhance the dispersion of the nanoparticles.

The PHBV-TFE solutions containing the nanoparticles were electrospun in a Fluidnatek^®^ LE-10 from Bioinicia S.L. (Valencia, Spain). The device operated under a steady flow rate using a multineedle injector, with horizontal scanning movement towards a metallic collector. The process was carried out using a flow rate of 6 mL/h, a needle-to-collector distance of 15 cm, and a voltage of 15 kV. The processing time was 2 h under controlled environmental conditions of 25 °C and 40% RH. The obtained electrospun fibers were maintained at 25 °C and 0% RH until further analysis.

### 2.3. Electrospun Films

The thermal post-treatment was carried out with a 4122 hot-plates press (Carver Inc., Wabash, IN, USA). The post-treatment was applied to the electrospun mats below the melting temperature (T_m_) of the biopolymer, at 160 °C for 10 s without pressure. This low temperature also avoids the thermal degradation of the polymer. The final samples showed a mean thickness between 70 and 80 µm.

### 2.4. Film Characterization

#### 2.4.1. Morphology

The PHBV electrospun fibers and their films loaded with CeO_2_NPs were analyzed by scanning electron microscopy (SEM) in a Hitachi S-4800 (Tokyo, Japan). Previous to SEM observation, samples were coated with gold–palladium for 3 min under vacuum conditions. SEM images were acquired at 10 kV. The cross section of the films was also performed. For that, the obtained films were immersed in liquid nitrogen and cryo-fractured.

Particle morphology as well as size of CeO_2_NPs loaded in electrospun PHBV fibers were characterized directly by TEM Hitachi HT7700 (Tokyo, Japan). Previously, samples were deposited onto clamping holders. TEM images were acquired at 100 kV. Image J Launcher v1.41 software (National Institutes of Health, Bethesda, USA) was used to determine the size of the nanoparticles along with the average fiber diameter using at least 20 images.

#### 2.4.2. Transparency

A UV4000 spectrophotometer (Dinko Instruments, Barcelona, Spain) was used to evaluate the light transmission of the films loaded with the nanoparticles. Samples of 50 mm × 30 mm were used to quantify the light absorption at wavelengths from 200 to 700 nm. The transparency value (T) was determined according to Equation (1), and the opacity value (O) was calculated according to Equation (2) [41]:

(1) $$\;T=\frac{A_{600}}{L}$$

(2) $$O=A_{500}\;L$$

A_500_ is the absorbance of the sample at 500 nm, whereas A_600_ is the absorbance of the sample at 600 nm. L corresponds to the thickness of the film (mm).

#### 2.4.3. Color

A Chroma Meter CR-400 (Konica Minolta, Tokyo, Japan) was employed to characterize the color of the films. The illuminant D65 was used. The color difference (∆E) was determined according to Equation (3):

(3) $$\Delta E={\left[{\left(\Delta L^{*}\right)}^{2}+{\left(\Delta a^{*}\right)}^{2}+{\left(\Delta b^{*}\right)}^{2}\right]}^{0.5}$$

where L* denotes the luminance (black to white), a* designates the change between green and red, and b* is the change from blue to yellow; ΔL*, Δa*, and Δb* corresponded to the differences between the brightness and color parameters of the PHBV films containing CeO_2_NPs and the values of the reference film (neat PHBV) (a* = 0.74, b* = −0.41, L* = 90.44) [42]. The color difference is considered unnoticeable if ΔE < 1. Color differences for ΔE ≥ 1 and < 2 are only detected by experienced personnel. Color differences for values of ΔE* ≥ 2 and < 3.5 can be detected by an inexperienced observer. Color difference for ΔE ≥ 3.5 and < 5 are clearly noticeable, and different colors are detected when ΔE ≥ 5 [43]. Tests were carried out in triplicate.

#### 2.4.4. X-ray Diffraction Analysis

An AXS D4 Endeavour diffractometer (Bruker Corporation, Billerica, MA, USA) was used to analyze the CeO_2_NPs and fiber samples by wide angle X-ray diffraction (WAXD). The analyses were performed at room temperature, in reflection mode with an incident CuKα radiation (k = 1.5406 Å); the generator was set to 40 kV, the filament current to 40 mA, and scattering angles (2θ) between 2 and 90° were used. Peak analysis was performed with the Igor Pro software using a Gaussian function to fit the data.

#### 2.4.5. Attenuated Total Reflection—Fourier Transform Infrared Spectroscopy (ATR-FTIR)

ATR-FTIR spectra were acquired by using the ATR sampling accessory Golden Gate (Specac Ltd., Orpington, UK) coupled to the Tensor 37 FTIR device (Bruker, Ettlingen, Germany). Spectra were obtained within the wavenumber range 4000–600 cm^−1^ by averaging 20 scans at 4 cm^−1^ resolution. Analysis of spectral data was performed using the OPUs 4.0 data collection software program (Bruker, Ettlingen, Germany).

#### 2.4.6. Thermal Analysis

The thermal transitions were studied with a DSC-8000 analyzer from PerkinElmer, Inc. (Waltham, MA, USA), coupled to a cooling accessory Intracooler 2 also from PerkinElmer, Inc. Samples followed a thermal sequence as follows: a first ramp from −30 °C to 190 °C, then a cooling step to −30 °C with heating and cooling rates of 10 °C/min. The measurements were performed under a nitrogen atmosphere using a flow rate of 20 mL/min. Sample weight was around 3.0 mg, using an empty aluminum pan as reference. Calibration was performed using an indium sample. Measurements were performed, at least, in duplicate. Thermograms were analyzed using the Pyris Manager software (PerkinElmer, Inc., Waltham, MA, USA).

A 550-TA Instruments Thermogravimetric Analyzer (New Castle, DE, USA) was used to perform the thermogravimetric analysis (TGA) between 25 °C to 700 °C, at a heating rate of 10 °C/min under a nitrogen atmosphere. The obtained data were analyzed by means of the TA analysis software. Measurements were carried out in triplicate.

#### 2.4.7. Oxygen Scavenging Capacity

The activity of the fibers and films containing CeO_2_NPs as oxygen scavengers were characterized using an OXY-4 mini device (PreSens Precision Sensing GmbH, Regensburg, Germany). For the measurements, 50 cm^3^ Schleck round-bottom flasks (VidraFoc S.A., Barcelona, Spain) with a polytetrafluoroethylene (PTFE) stopcock were used. An O_2_-sensitive sensor spot (PSt3, detection limit 15 ppb, 0–100% oxygen, PreSens) was glued onto the inner wall of the flasks. Then, 5 × 5 cm^2^ samples were put inside the flasks, flushed for three minutes with 100 vol.% N_2_, and then the gas mixture containing 4 vol. % oxygen, 2 vol. % hydrogen, and 94 vol. % nitrogen (Abelló Linde, S.A. Barcelona, Spain) was injected for 1 min at 1 bar. The oxygen concentration analysis inside the flask in function of time was performed via the fluorescence decay method by means of the OXY-4 mini (PreSens). Measurements were carried out at a temperature of 23 °C and 100% RH.

#### 2.4.8. Mechanical Test

Mechanical properties were determined with a universal mechanical testing device (AGS-X 500N, Shimadzu Corp. Kyoto, Japan) at room temperature. The load cell was 1 kN, and the cross-head speed was 10 mm/min. Tests were performed following the ASTM D638 (Type IV) standard. Samples were shaped into dumbbell specimens. At least six specimens were analyzed for each sample. Tensile modulus (E), tensile strength at yield (σ_y_), elongation at break (ε_b_), and toughness (T) were determined from the stress–strain curves calculated from the force–distance data.

#### 2.4.9. Barrier Properties

The water vapor permeability (WVP) of the film samples was obtained with a gravimetric method ASTM E96-95. For this, 5 mL of distilled water was placed inside a Payne permeability cup (diameter of 3.5 cm) (Elcometer Sprl, Hermallesous-Argenteau, Belgium). The films were exposed to 100% RH on one side. The samples were kept inside a desiccator at 0% RH and 25 °C. Aluminum films were employed as control samples to evaluate the solvent loss through the sealing. An analytical balance (±0.0001 g) was used to determine the weight loss of the cups. WVP was determined from the regression analysis of weight loss data versus time, and the weight loss was corrected taking into account the small losses through the sealing. The permeability was obtained taking into account the permeance and the average film thickness.

Following a similar methodology, limonene permeability (LP) was evaluated with Payne permeability cups with 5 mL of D-limonene (25 °C and 40% RH). The permeation rate of limonene vapor (LPRT) was calculated from the steady-state permeation slopes, and the weight loss was corrected with the loss through the sealing. In addition, the average film thickness was taken into consideration for the LP determination. The analyses were performed in triplicate.

The oxygen permeability coefficient was calculated from oxygen transmission rate results obtained by means of an Oxygen Permeation Analyzer M8001 from Systech (Illinois, UK) and according to the ASTM D3985-05 standard. Experiments were performed at 23 °C and 60% RH. Samples were flushed with nitrogen before being exposed to an oxygen flow of 10 mL min^−1^; 5 cm^2^ was the exposure area for each sample during the test. Film thickness and gas partial pressure were taken into consideration to calculate the oxygen permeability. The analyses were conducted in triplicate.

### 2.5. Antimicrobial Activity

The antimicrobial properties of the PHBV films loaded with CeO_2_NPs were evaluated following the Japanese Industrial Standard (JIS) Z 2801:2010. Common food bacteria, specifically *S. aureus* CECT240 (ATCC 6538P) and *E. coli* CECT434 (ATCC 25922) strains, were provided by the Spanish Type Culture Collection (CECT) (Valencia, Spain). They were reconstituted and kept in phosphate-buffered saline (PBS) with 10 wt.% tryptic soy broth (TSB) and 10 wt.% glycerol at −80 °C. A loopful of bacteria was relocated to 10 mL of TSB and incubated at 37 °C for 24 h. A 100 μL aliquot from the bacterial culture was again relocated to TSB and grown at 37 °C to the mid-exponential phase of growth, when approximately 5 × 10^5^ colony-forming units (CFU)/mL of culture were obtained.

Film samples containing CeO_2_NPs were cut in squares 1.5 cm × 1.5 cm. Additionally, a polyethylene film was taken as the control film because it shows no antimicrobial activity. To determine the antimicrobial activity, a suspension of *S. aureus* and *E. coli* was deposited on the film samples and incubated for 24 h at 24 °C in at least, 95% RH. The bacteria were then recovered with PBS, 10-fold serially diluted, and incubated at 37 °C for 24 h to quantify the number of viable bacteria by plate count. The antimicrobial reduction (R) was determined by means of Equation (4):

(4) $$R=\left[\mathrm{Log}\left(\frac{B}{A}\right)-\left(\frac{C}{A}\right)\right]=\mathrm{Log}\left(\frac{B}{C}\right)$$

where A is the average of the number of bacterial counts for the control sample immediately after inoculation. Alternatively, B is known as the average of the number of bacterial counts for the control sample after 24 h, whilst C is the average of the number of bacterial counts for the film sample after 24 h. Antimicrobial activity was assessed taking into account that values of R < 0.5 are considered non-significant, values of R ≥ 0.5 and < 1 are considered slight, values of R ≥ 1 and < 3 are significant, and values of R ≥ 3 mean strong reduction.

### 2.6. Antioxidant Activity

The DPPH method was run to determine the antioxidant effect of the CeO_2_NPs, CTAB, fibers, and films. Approximately 10 mg of the sample was used and then 3 mL of the DPPH stock solution (0.04 g/L in aqueous methanol) wase added. DPPH solution was used as a control. Methanol was used as blank. Samples were kept at room temperature for 24 h in the dark. Subsequently, the absorbance of the samples was evaluated in a UV 4000 spectrophotometer (Dinko Instruments, Barcelona, Spain) at a wavelength of 517 nm. Equation (5) was used to calculate the percentage of DPPH inhibition [44].

(5) $$\mathrm{DPPH}\;\mathrm{Inhibition}\;\left(\%\right)=\frac{A_{\mathrm{Control}}-\left(A_{\mathrm{sample}}-A_{\mathrm{blank}}\right)}{A_{\mathrm{control}}}*100\;$$

where A_control_ is the absorbance of the DPPH solution, A_blank_ is the absorbance of the methanol, and A_sample_ is the absorbance of the test sample.

### 2.7. Statistical Analysis

Statistically significant results obtained for the different samples were assessed via the analysis of variance (ANOVA) with a 95% significance level (*p* ≤ 0.05) and a multiple comparison test (Tukey) using the software OriginPro8 (OriginLab Corporation, Northampton, MA, USA).

## 3. Results and Discussion

### 3.1. Morphological Characterization

**Figure 1:**
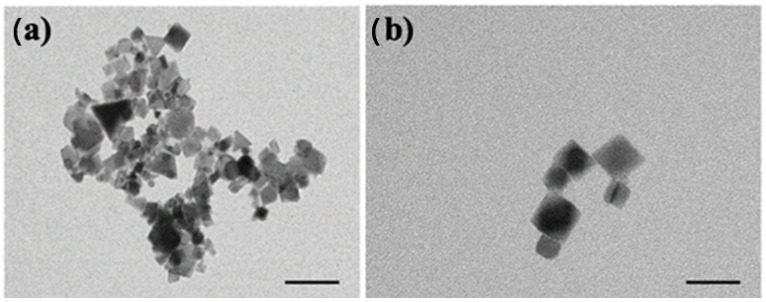
Images of cerium oxide nanoparticles (CeO_2_NPs) taken by transmission electron microscopy (TEM): (**a**) scale 50 nm; (**b**) scale bar 20 nm.

First, the morphological characterization of the CeO_2_NPs was performed by TEM. Figure 1a illustrates that the morphology of the nanoparticles is made of cubes of approximately 20 × 20 nm^2^. The vast majority of CeO_2_NPs had sizes ranging from 15–25 nm. The observed morphology was in agreement with observations by Salarizadeh et al. [45], who reported CeO_2_ nanoparticles with average sizes of ca. 25 nm.

**Figure 2:**
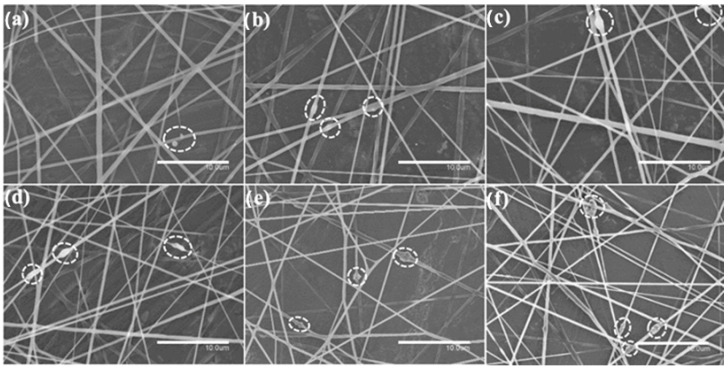
Comparison of the morphology of the electrospun poly(3-hydroxybutyrate-co-3-hydroxyvalerate) (PHBV) fibers: (**a**) 0.5 wt.% CeO_2_NPs; (**b**) 1.0 wt.% CeO_2_NPs; (**c**) 1.5 wt.% CeO_2_NPs; (**d**) 2.0 wt.% CeO_2_NPs; (**e**) 5.0 wt.% CeO_2_NPs; (**f**) 10.0 wt.% CeO_2_NPs. Micrographs were taken by scanning electron microscopy (SEM). Scale bar corresponds to 10 µm. Dotted circles highlight the presence of beads and aggregates within the fibers.

**Figure 3:**
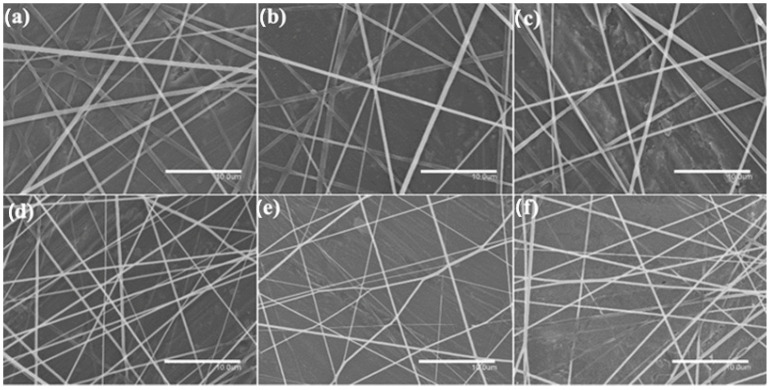
Comparison of the morphology of the electrospun poly(3-hydroxybutyrate-co-3-hydroxyvalerate) (PHBV) fibers: (**a**) 0.5 wt.% CeO_2_NPs + CTAB; (**b**) 1.0 wt.% CeO_2_NPs + CTAB; (**c**) 1.5 wt.% CeO_2_NPs + CTAB; (**d**) 2.0 wt.% CeO_2_NPs + CTAB; (**e**) 5.0 wt.% CeO_2_NPs + CTAB; (**f**) 10.0 wt.% CeO_2_NPs + CTAB. Micrographs were taken by scanning electron microscopy (SEM). Scale bar 10 µm.

Figure 2 and Figure 3 show the morphology of the electrospun PHBV fibers containing CeO_2_NPs and CeO_2_NPs + CTAB, respectively. The incorporation of CTAB enhanced the distribution of CeO_2_NPs inside the PHBV fibers when comparing Figure 2 and Figure 3. The PHBV fibers containing CeO_2_NPs without CTAB (Figure 2) presented beads and aggregates. It can be seen that the CeO_2_NPs resulted in larger fiber diameter in the 0.58–0.65 μm range and also led to the formation of spindle-type beads. This phenomenon is seen in Figure 2d–f, which corresponds to 2 wt.%, 5 wt.%, and 10 wt.% CeO_2_NPs within the PHBV matrix, respectively. This observation suggests that some agglomeration of CeO_2_NPs may occur in some fiber regions. On the other hand, the electrospun PHBV fibers containing CeO_2_NPs + CTAB gathered in Figure 3 showed a smooth, homogeneous, and bead-free morphology, demonstrating the advantage of surfactants in enhancing nanoparticle dispersion. Analysis on fiber diameter revealed a 0.50–0.60 μm size, being the thinnest fibers containing the highest concentration of CeO_2_NPs (Figure 3d–f). It has been previously reported that the diameter of nanofibers diminishes when adding surfactants and nanoparticles in a polymer solution due to an effect on solution parameters (i.e., surface tension or conductivity) that in turn affects the stretching forces of the jet, generating fibers with smaller diameters [46]. For instance, the conductivity of solutions containing 1.5 wt.% and 5.0 wt.% CeO_2_NPs increased significantly with the addition of CTAB from 7.07 to 61.98 µS/cm and from 8.57 to 62.84 µS/cm, correspondingly. All the electrospun fibers presented here decreased in diameter when CeO_2_NPs were added compared to neat PHBV fibers, which showed diameters of ~0.78 µm according to our previous studies [47,48]. This phenomenon could be ascribed to the change in charge density and conductivity as the concentration of CeO_2_NPs increased [49]. Cherpinski et al. [33] observed that the incorporation of surfactants, i.e., CTAB and TEOS, successfully enhanced the distribution of PdNPs in the PHB fibers, similar to the observation reported in this work.

**Figure 4:**
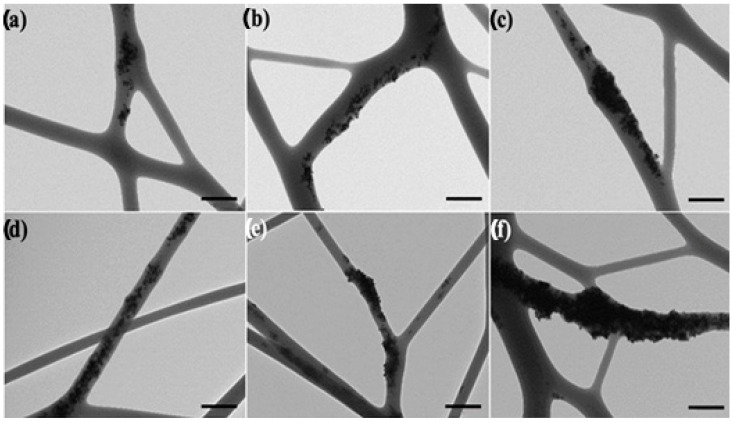
Comparison of the nanoparticles dispersion within the electrospun poly(3-hydroxybutyrate-co-3-hydroxyvalerate) (PHBV) fibers: (**a**) 0.5 wt.% CeO_2_NPs; (**b**) 1.0 wt.% CeO_2_NPs; (**c**) 1.5 wt.% CeO_2_NPs; (**d**) 2.0 wt.% CeO_2_NPs; (**e**) 5.0 wt.% CeO_2_NPs; (**f**) 10.0 wt.% CeO_2_NPs. Micrographs were taken by transmission electron microscopy (TEM). Scale bar 400 nm.

**Figure 5:**
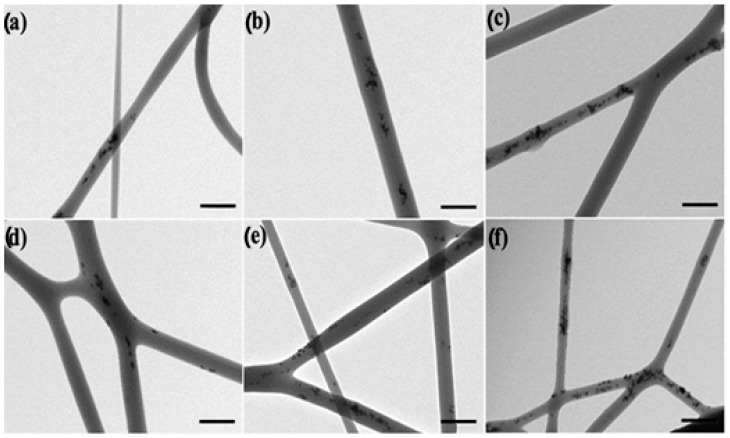
Comparison of the nanoparticles dispersion within the electrospun poly(3-hydroxybutyrate-co-3-hydroxyvalerate) (PHBV) fibers: (**a**) 0.5 wt.% CeO_2_NPs + CTAB; (**b**) 1.0 wt.% CeO_2_NPs + CTAB; (**c**) 1.5 wt.% CeO_2_NPs + CTAB; (**d**) 2.0 wt.% CeO_2_NPs + CTAB; (**e**) 5.0 wt.% CeO_2_NPs + CTAB; (**f**) 10.0 wt.% CeO_2_NPs + CTAB. Micrographs were taken by transmission electron microscopy (TEM). Scale bar 400 nm.

Figure 4 and Figure 5 showed the TEM images of the electrospun fibers in order to assess the dispersion and distribution of the CeO_2_NPs inside the PHBV fibers, without and with CTAB, respectively. The CeO_2_NPs were successfully incorporated within the PHBV fibers by means of the electrospinning process. However, as the nanoparticle concentration increased, so did their agglomeration. Figure 4a–f show the PHBV fibers containing 0.5, 1, 1.5, 2, 5, and 10 wt.% of CeO_2_NPs, which present clear agglomeration features, whereas the fibers containing 0.5, 1, 1.5, 2, 5, and 10 wt.% CeO_2_NPs + CTAB (Figure 5a–f) present a better CeO_2_NPs dispersion due to the surface activity of CTAB. Despite the fact that CTAB significantly improved the nanoparticles dispersion, these were seen heterogeneously distributed within the PHBV matrix of the fibers. This morphology further confirms the observations made by SEM in the PHBV fibers, demonstrating the successful enhancement in dispersion and distribution of the CeO_2_NPs within the PHBV matrix as a result of adding the CTAB surfactant.

**Figure 6:**
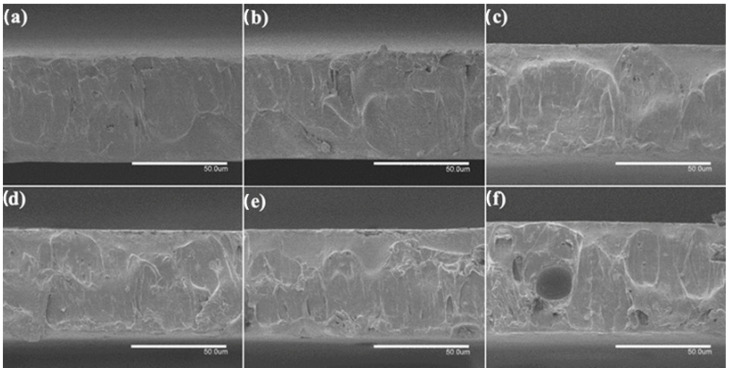
Comparison of the cross-section morphology for the electrospun poly(3-hydroxybutyrate-co-3-hydroxyvalerate) (PHBV) films: (**a**) 0.5 wt.% CeO_2_NPs; (**b**) 1.0 wt.% CeO_2_NPs; (**c**) 1.5 wt.% CeO_2_NPs; (**d**) 2.0 wt.% CeO_2_NPs; (**e**) 5.0 wt.% CeO_2_NPs; (**f**) 10.0 wt.% CeO_2_NPs. Images were taken by scanning electron microscopy (SEM). Scale bar corresponds to 50 µm.

**Figure 7:**
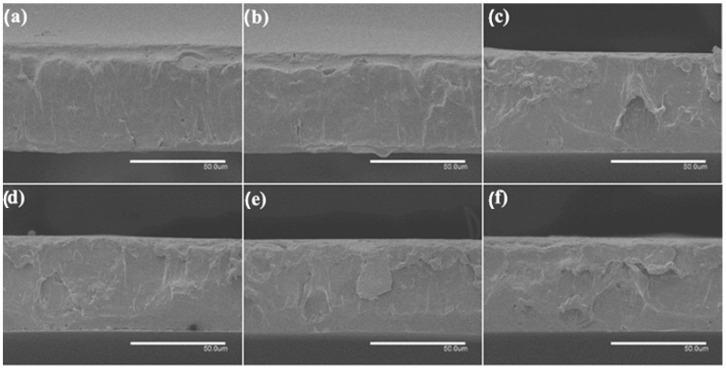
Comparison of the cross-section morphology for the electrospun poly(3-hydroxybutyrate-co-3-hydroxyvalerate) (PHBV) films: (**a**) 0.5 wt.% CeO_2_NPs + CTAB; (**b**) 1.0 wt.% CeO_2_NPs + CTAB; (**c**) 1.5 wt.% CeO_2_NPs + CTAB; (**d**) 2.0 wt.% CeO_2_NPs + CTAB; (**e**) 5.0 wt.% CeO_2_NPs + CTAB; (**f**) 10.0 wt.% CeO_2_NPs + CTAB. Images were taken by scanning electron microscopy (SEM). Scale bar 50 µm.

A thermal post-treatment at 160 °C was applied to the electrospun fiber mats to be turned into films. To analyze the internal morphology of these films, they were cryo-fractured with liquid N_2_ and observed by SEM. Figure 6 and Figure 7 show SEM images of the cross sections of the PHBV films loaded with CeO_2_NPs and with CTAB, correspondingly. The pure PHBV film presented a thickness of ~80 μm, as in our previous works [47,48]. The CeO_2_NPs-containing PHVB films (Figure 6) showed a uniform and non-porous structure with thicknesses in the 75–85 μm range; only a few pores were observed in the PHBV film containing 10 wt.% CeO_2_NPs in its cross section, probably due to the most likely detachment of filler agglomerates. The PHBV films loaded with CeO_2_NPs + CTAB showed similar morphologies as shown in Figure 7. These films showed more uniform, smooth, and homogeneous surfaces compared to the films without CTAB with thicknesses in the 70–81 μm range, which is in agreement with the fibers studied by SEM and TEM, in which the good dispersion of the nanoparticles due to CTAB could be inferred. The incorporation of CeO_2_NPS also slightly augmented the film thicknesses up to ~85 μm in all film samples, which may be related to the restrictions of the fibers for its reorganization due to the presence of the nanoparticles whilst annealing. Several research studies have reported increased film thickness and the appearance of cracks and pores since the nanoparticles tend to disrupt the homogeneous matrix within the polymer fibers [34,41,47].

### 3.2. Optical Characterization of the Electrospun Films

**Figure 8:**
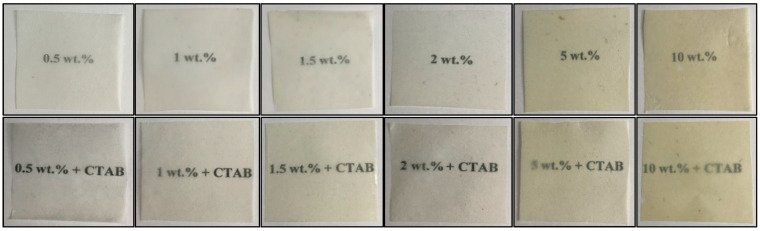
Comparison of the visual aspect of electrospun poly(3-hydroxybutyrate-co-3-hydroxyvalerate) (PHBV) films loaded with cerium oxide nanoparticles (CeO_2_NPs) and hexadecyltrimethylammonium bromide (CTAB).

The electrospun PHBV films containing CeO_2_NPs are shown in Figure 8. A macroscopic inspection was used to evaluate their contact transparency, and Table 1 gathers the variations on the color coordinates (L*, a*, b*) and on the values of ΔE, T, and O due to the incorporation of CeO_2_NPs. The optical characterization of the pure PHBV film was also presented for the purpose of comparison. All PHBV films showed contact transparency, although films developed some yellow colorification when the CeO_2_NPs were added, reducing brightness (L*) and increasing opacity (O), which was corroborated by the rise in the b* coordinate. The consequences of the presence of the nanoparticles on color modification were related to nanoparticle concentration; for instance, concentrations of 5 and 10 wt.% CeO_2_NPs showed values of ΔE* ≥ 5, meaning that an observer can distinguish different colors [43], whereas films with lower concentrations of nanoparticles showed a slight color change. Thus, films containing 1.5 and 2 wt.% CeO_2_NPs exhibited a major color difference with ΔE* ≥ 3.5 and < 5, and the films containing 1 wt.% CeO_2_NPs a exhibited a color change with ΔE* ≥ 2 and < 3.5, where an inexperienced observer can notice the color difference. For films containing 0.5 wt.%, the color difference was unnoticeable with ΔE* < 1, similar to the neat PHBV.

Neat PHBV showed transparency and opacity values similar to the values reported by Figueroa et al. for the same biopolymer [41]. However, other polymeric films made of electrospun PCL showed reduced transparency in comparison to neat PHBV [50]. The presence of the CeO_2_NPs caused light scattering, reducing the ability of transmission among visible and UV light of the films. Particularly, the films containing 5 and 10 wt.% CeO_2_NPs reduced the transparency properties and augmented the opacity of the films. This phenomenon was also observed for other metallic nanofillers such as ZnO [41]. However, this property may also be a preferred characteristic for some food packaging solutions to avoid oxidative reactions of lipids, carbohydrates, and proteins due to the action of ultraviolet light. These results agree with previous studies indicating that the incorporation of nanofillers increased the opacity, a*, and b* values, and decreased L* and the transparency results of biodegradable films [51,52].

### 3.3. X-ray-Diffraction (XRD) of the Electrospun Fibers

**Figure 9:**
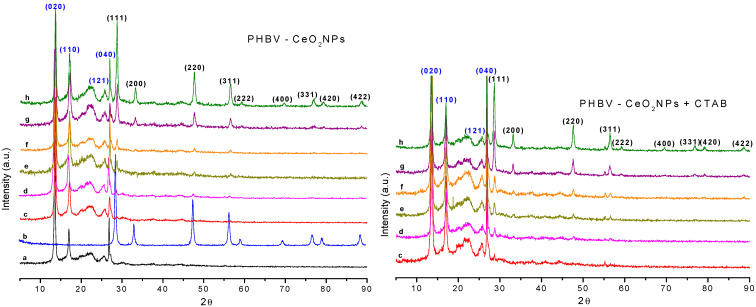
Diffractograms of cerium oxide nanoparticles (CeO_2_NPs), and electrospun poly(3-hydroxybutyrate-co-3-hydroxyvalerate) (PHBV) fibers loaded with CeO_2_NPs: (**a**) neat PHBV; (**b**) CeO_2_NPs; (**c**) 0.5 wt.% CeO_2_NPs; (**d**) 1.0 wt.% CeO_2_NPs; (**e**) 1.5 wt.% CeO_2_NPs; (**f**) 2.0 wt.% CeO_2_NPs; (**g**) 5.0 wt.% CeO_2_NPs; (**h**) 10.0 wt.% CeO_2_NPs.

Diffractograms of CeO_2_NPs and electrospun PHBV fibers containing CeO_2_NPs and CTAB are displayed in Figure 9. The peaks of the neat CeO_2_NPs sample were ascribed to the diffraction planes (Miller indices) of (111), (200), (220), (311), (222), (400), (331), (420), and (422) at 2θ values of 28.70°, 33.14°, 47.59°, 56.48°, 59.11°, 69.63°, 76.79°, 79.21°, and 88.61°, respectively. These planes belong to the cubic phase of CeO_2_; the orderly peaks arrangement highlights a high crystallinity of the CeO_2_NPs. The same peaks for CeO_2_NPs were observed by Wang et al. [53]. Moreover, Youn et al. [54] reported the predominant peaks of CeO_2_NPs assigned to the (111), (200), (220), (311), (222), (400), and (311) planes, indicating that CeO_2_NPs were ascribed to the pure fluorite cubic structures of CeO_2_. The neat PHBV showed the characteristic peaks at 2θ values of 13.55°, 16.99°, 25.64°, and 26.86°. These peaks are ascribed to the diffraction planes of (020), (110), (121), and (040), respectively [41]. The crystalline lattice parameters are in concordance with the parameters previously described for PHBV3%, PHBV12%, and PHBV18% 3HV [55]. It is well-known that PHBV copolymers with fractions of 3HV lower than 40 mol% crystallize within the PHB crystalline lattice and exhibit the same PHB homopolymer diffractograms [56,57,58]. In this regard, the PHBV here used with 2 mol% 3HV content presented the same orthorhombic crystal structure as the homopolymers PHB [59]. The CeO_2_NPs-loaded PHBV samples showed peaks that confirmed the presence of the nanoparticles in the PHBV fibers with a lower relative intensity because of the dilution effect.

### 3.4. Attenuated Total Reflection-Fourier Transform Infrared Spectroscopy (ATR-FTIR) of the Electrospun Fibers

**Figure 10:**
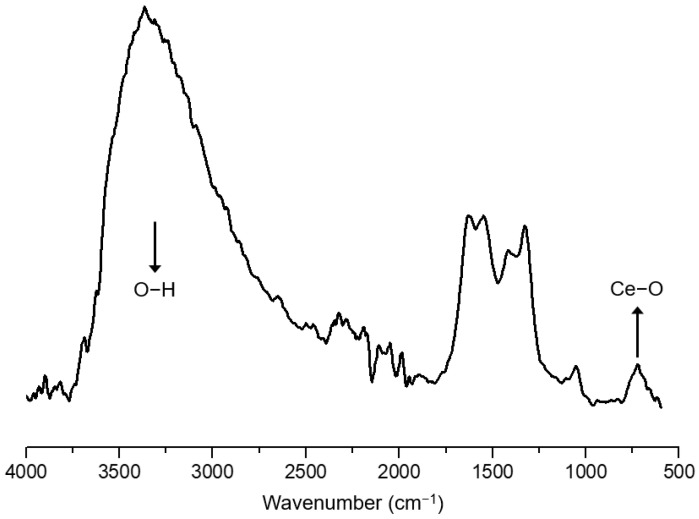
ATR-FTIR spectra of cerium oxide nanoparticles (CeO_2_NPs). The spectra were acquired between a 4000 and 600 cm^−1^ wavenumber.

**Figure 11:**
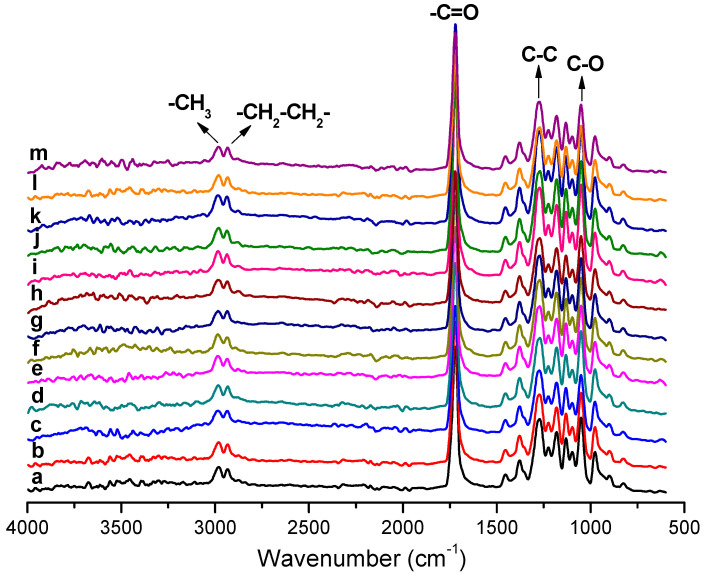
ATR-FTIR spectra of electrospun poly(3-hydroxybutyrate-co-3-hydroxyvalerate) (PHBV) fibers loaded with cerium oxide nanoparticles (CeO_2_NPs): (**a**) neat PHBV; (**b**) 0.5 wt.% CeO_2_NPs; (**c**) 0.5 wt.% CeO_2_NPs + CTAB; (**d**) 1.0 wt.% CeO_2_NPs; (**e**) 1.0 wt.% CeO_2_NPs + CTAB; (**f**) 1.5 wt.% CeO_2_NPs; (**g**) 1.5 wt.% CeO_2_NPs + CTAB; (**h**) 2.0 wt.% CeO_2_NPs; (**i**) 2.0 wt.% CeO_2_NPs + CTAB; (**j**) 5.0 wt.% CeO_2_NPs; (**k**) 5.0 wt.% CeO_2_NPs + CTAB; (**l**) 10.0 wt.% CeO_2_NPs; (**m**) 10.0 wt.% CeO_2_NPs + CTAB. The spectra were acquired between 4000 and 600 cm^−1^ wavenumber.

ATR-FTIR absorption spectra for CeO_2_NPs (Figure 10) as well as electrospun PHBV fibers containing CeO_2_NPs and CTAB (Figure 11) were scanned between 4000 and 600 cm^−1^. The ATR-FTIR spectra of pure CeO_2_NPs suggested a pronounced peak at 3344 cm^−1^ ascribed to the O–H vibration of sorbed water on the CeO_2_ surface [60]. The band below 700 cm^−1^ is assigned to the Ce–O stretching mode vibration of nCeO_2_ [61]. Figure 11 shows the typical vibrational bands arising from the functional groups of the PHBV. The bands from 2987 to 2932 cm^−1^ are attributed to the asymmetric stretching mode of the methyl (–CH_3_) and antisymmetric stretching mode of methylene (–CH_2_), respectively [62]. The band at 1720 cm^−1^ belongs to the carbonyl stretching band (C=O). The stretching vibration of the C–O groups is ascribed to the peak from 1447 and 1000 cm^−1^. The strong vibration band at 1275 cm^−1^ is assigned to the C–C group and at 1053 cm^−1^ to the C–O group. The absorption bands at 977, 891, and 820 cm^−1^ belonged to the C–C groups [63]. Moreover, the absorbance peaks at 1093 and 1184 cm^−1^ were ascribed to the stretching vibration of ether (C–O–C) [64,65,66]. The spectra of the PHBV containing CeO_2_NPs also showed similar characteristic peaks as for pure PHBV, demonstrating that the incorporation of CeO_2_NPs + CTAB did not affect the PHBV spectra. As reported by other authors before, changes in the PHBV spectra occur primarily in the most prominent bands between 1720 and 1740 cm^−1^ region (C=O); band shifts in this region have been attributed to changes in crystallinity [67], as well as to nanoparticle–matrix interactions [68,69]. Nevertheless, for the PHBV prepared here, the nCeO_2_-specific bands were not observed because of the low concentration of CeO_2_NPs within the PHBV matrix, and band shifts in the above-cited regions were not observed, suggesting no measurable interactions between the filler and the biopolymer [70].

### 3.5. Thermal Properties

#### 3.5.1. Differential Scanning Calorimetry (DSC) of the Electrospun Fibers

DSC was performed to discern the main thermal events of electrospun PHBV fibers containing CeO_2_NPs and CTAB. Results are shown in Table 2. In the first heating, the pure PHBV showed a single melting phenomenon at 169.46 °C. The melting point for all PHBV fibers containing CeO_2_NPs slightly increased up to 172.66 °C for the sample with a 5 wt.% CeO_2_NPs. Nevertheless, the T_m1_ values for samples containing CeO_2_NPs + CTAB slightly decreased up to 162.85 °C for the sample with a 10 wt.% CeO_2_NPs. On the other hand, the enthalpy of melting (ΔH_m1_) of PHBV was reduced by CeO_2_NPs + CTAB with values in the 65.10–77.98 J/g range. All samples showed a unique crystallization peak during cooling. For instance, the crystallization temperature (T_c_) of the pure PHBV was 117.35 °C. Samples containing CeO_2_NPs slightly augmented the crystallization temperature of PHBV up to 119.60 °C for the sample containing a 10 wt.% CeO_2_NPs, indicating a nucleating effect of the PHBV matrix, whereas the samples with CeO_2_NPs and CTAB slightly decreased the T_c_ up to a value of 116.05 °C for samples with a concentration of 10 wt.% CeO_2_NPs. This slight change in crystallization temperature can be attributed to the dispersing effect of the surfactant, suggesting that a better dispersion of the CeO_2_NPs can impair to some extent the packing of the PHBV chains during cooling [33]. During the second heating, the melting point (T_m2_) for all samples shifted slightly towards a higher temperature with values in the 172.53–176.80 °C range. The enthalpy of melting (ΔH_m2_) in the second heating showed somewhat lower values compared to the first heating with values in the 63.32–73.49 J/g range. This thermal behavior is in agreement with Augustine et al. [70] who communicated that CeO_2_ loadings did not lead to a considerable change in the thermal properties of PHBV, such as its melting point or its crystallization temperature, but did cause a slight variation in the enthalpies of fusion and crystallization of the developed materials. Similar observations were also made before for other nanofillers, such as ZnO [71], Ag [72], CuO [73], and mesoporous silica [47].

#### 3.5.2. Thermogravimetric Characterization of the Electrospun Fibers

Thermogravimetric analysis was run from 25 °C to 700 °C. Table 3 gathers the temperature for 5% weight loss (T_5%_), the degradation temperature (T_deg_), and the residual mass at 700 °C obtained by TGA. Data shown in this table shows that CeO_2_NPs were thermally stable up to 700 °C, displaying a discrete weight loss (∼3.56%) below 500 °C attributed to the evaporation of sorbed water. This thermal stability is characteristic of metal oxide nanoparticles [74]. The pure PHBV showed a Tdeg of 278.7 °C and a residual mass of 1.13%. Similar results were reported for PHBV-based materials with values between 270 and 290 °C [47,48,64,75]. The presence of the CeO_2_NPs reduced the temperature for 5% weight loss and the T_deg_ compared to pure PHBV. This decrease in onset degradation temperatures may be attributed to the CTAB decomposition and to the high thermal conductivity properties of the CeO_2_NPs. It has been reported that the addition of metallic nanoparticles to polymeric films can lead to a decrease of the degradation temperature; however, the effect of the filler depends on the type, content, interfacial interaction, and the degree of dispersion and distribution of the particles in the polymer matrix [34]. Additionally, the obtained results prove that CeO_2_ nanoparticles are able to scavenge O_2_ at room temperature under the presence of humidity and hydrogen in the headspace gas mixture.

Gofman et al. also observed a decrease in the degradation temperature when preparing bacterial cellulose films containing CeO_2_ nanoparticles. Additionally, the degradation temperature decreased as the content of CeO_2_ nanoparticles increased [76]. Brito et al. also observed that the addition of metallic fillers decreased the thermal stability of the polymeric matrix [77].

Residual mass was between 1.03 and 17.22% for all samples, which increased with the CeO_2_NPs’ concentration due to their non-degradable nature. Moreover, the samples with CTAB showed a general trend of somewhat higher residual mass compared to the samples without CTAB, possibly attributed to a potential catalytic effect that would be promoting the formation of higher char residues during the degradation stage [78]. Castro-Mayorga et al. [75] also observed a reduction of T_deg_ for PHBV containing ZnO.

### 3.6. Oxygen Scavenging Capacity of Electrospun Fibers and Films

**Figure 12:**
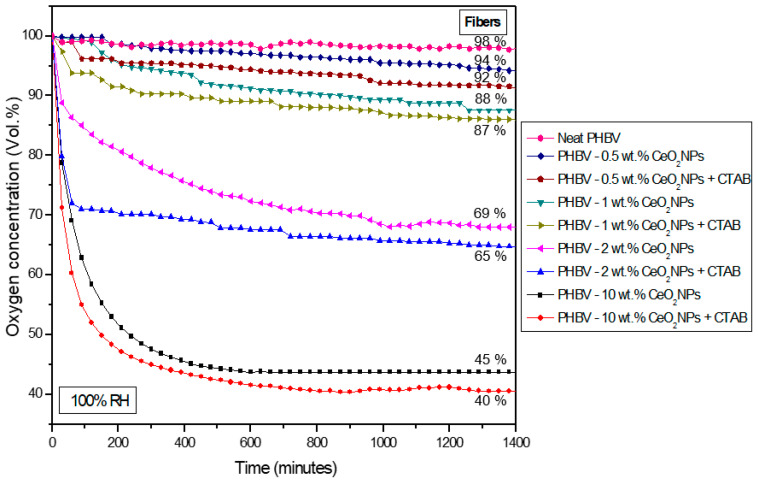
Headspace oxygen volume depletion of electrospun poly(3-hydroxybutyrate-co-3-hydroxyvalerate) (PHBV) fibers loaded with 0.5 wt.%, 1.0 wt.%, 2.0 wt.%, and 10.0 wt.% cerium oxide nanoparticles (CeO_2_NPs) with and without hexadecyltrimethylammonium bromide (CTAB). The samples were tested at 100% relative humidity (RH).

**Figure 13:**
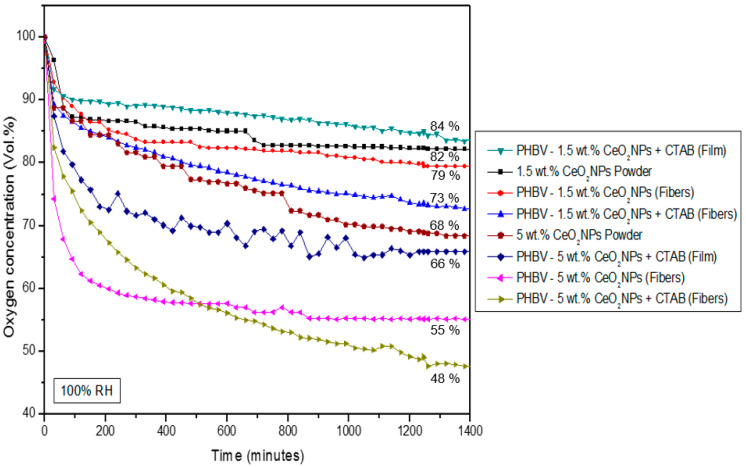
Headspace oxygen volume depletion of cerium oxide nanoparticles (CeO_2_NPs), electrospun poly(3-hydroxybutyrate-co-3-hydroxyvalerate) (PHBV) fibers, and films loaded with 1.5 wt.% and 5.0 wt.% cerium oxide nanoparticles (CeO_2_NPs) with and without hexadecyltrimethylammonium bromide (CTAB). The samples were tested at 100% relative humidity (RH).

The oxygen scavenger profile of the electrospun PHBV fibers and selected films containing CeO_2_NPs + CTAB was analyzed by determining the oxygen scavenging rate (OSR) with an initial oxygen concentration of 4.0% in the headspace of the Schleck flasks. The mechanism of action of oxygen scavengers is mainly associated with their ability to catalyze the oxidation of hydrogen, which can then remove residual oxygen in the packaging headspace [16]. Figure 12 and Figure 13 show the oxygen concentration depletion over time for a timeframe of 1400 min at 23 °C and 100% RH. Figure 12 shows that the neat PHBV fibers were not able to scavenge oxygen, while PHBV fibers containing CeO_2_NPs in the 0.5–10 wt.% concentrations range showed a reduction of headspace oxygen from 6% up to 60%. From Figure 13, it can be seen that free CeO_2_NPs in a powder form in similar quantities as used in the samples of 1.5 wt.% and 5 wt.% were able to reduce up to 17.8% and 31.6%, respectively, of the headspace oxygen. The PHBV fibers containing 1.5 wt.% and 5 wt.% CeO_2_NPs presented a depletion of 20.7% and 44.8%, respectively. The CTAB-containing PHBV/CeO_2_NPs fibers improved the oxygen scavenging activity, which further confirmed the surfactant-induced enhanced dispersion and distribution of the nanoparticles, as observed above by TEM, achieving a depletion of 27.4% (1.5 wt.% CeO_2_NPs) and 52.3% (5 wt.% CeO_2_NPs). However, when the selected fibers turned into films after the annealing process, the oxygen depletion decreased to 16.1% and 34.1% for PHBV films loaded with 1.5 wt.% and 5 wt.% CeO_2_NPs, correspondingly. This decrease in the OSR is associated with the lower surface-to-volume ratio of the films compared to the fibers because the annealing treatment reduced the interfiber porosity by a coalescence process. Regardless of this, the PHBV film containing 5 wt.% CeO_2_NPs presented significant oxygen scavenging capacity. Additionally, the obtained results prove that CeO_2_ nanoparticles are able to scavenge O_2_ at room temperature under the presence of humidity and some hydrogen in the headspace gas composition. These observations are in concordance with Cherpinski et al. [33,34] who studied the oxygen scavenging capacity of poly(3-hydroxybutyrate) (PHB) and polycaprolactone (PCL) biopolymers containing palladium nanoparticles (PdNPs) and surfactants prepared by electrospinning, also concluding that the fibers provided better oxygen scavenging performance than the annealed films.

### 3.7. Mechanical Properties of the Electrospun Films

Table 4 shows the mechanical characterization of the selected electrospun PHBV films loaded with CeO_2_NPs and CTAB. The pure PHBV film showed an E of 2394 MPa, a σ_y_ of 14.1 MPa, an ε_b_ of 1.01%, and a T of 0.09 mJ/m^3^. Similar mechanical values were observed by Melendez-Rodriguez et al. [47] for electrospun films of pure PHBV, where E was 1252 MPa, σ_y_ was 18.1 MPa, and ε_b_ was 2.4%. The addition of CeO_2_NPs into the PHBV matrix increased the mechanical values; at 1.5 wt.% CeO_2_NPs, the E value was 3309 MPa, σ_y_ was 26.9 MPa, ε_b_ was 1.22%, and T was 0.18 mJ/m^3^. At 5 wt.% CeO_2_NPs, the E value was 3546 MPa, σ_y_ was 27.52 MPa, ε_b_ was 1.19%, and T was 0.18 mJ/m^3^. This increase in mechanical properties can be ascribed to the nanofillers’ reinforcement effect, which may also be related to aspects, such as nanoparticle dispersion, nanofiller concentration, changes in polymer crystallinity, and the interfacial adhesion between the nanoparticles and the biopolymer matrix [79]. Similar reinforcing effects on the PHBV matrix were observed by Figueroa-Lopez et al. [41] when ZnONPs were incorporated. Ashori et al. [80] also observed that the incorporation of nanofillers, such as cellulose nanocrystals and aluminum oxide nanoparticles within the PHBV matrix, significantly increased the mechanical properties of PHBV composites.

### 3.8. Barrier Properties of the Electrospun Films

Table 5 gathers the permeability to water (WVP) and limonene (LP) vapors of the neat PHBV and the selected PHBV films loaded with CeO_2_NPs and CTAB. The water and limonene permeability values for neat PHBV were 5.34 × 10^−14^ and 26.8 × 10^−15^ kg·m·m^−2^·s^−1^·Pa^−1^, respectively [47]. The incorporation of CeO_2_NPs into PHBV diminished the permeability of both vapors. The PHBV films containing 1.5 wt.% CeO_2_NPs presented WPV and LP results of 1.58 × 10^−14^ and 6.71 × 10^−15^ kg·m·m^−2^·s^−1^·Pa^−1^, respectively. Regarding samples containing 5.0 wt.% CeO_2_NPs, the WVP and LP results were 2.68 × 10^−14^ and 8.23 × 10^−15^ kg·m·m^−2^·s^−1^·Pa^−1^, respectively. The reduction in WVP can be due to the presence of CeO_2_NPs into PHBV, which potentially sorbed water over the surface of the nanoparticles and further blocked water transport through the polymer matrix, due to a more tortuous path generated by the homogenized distribution of the nanoparticles [42,73]. The decrease in limonene permeability may be associated with a reduction in the sorption of limonene molecules by the PHBV film, where solubility plays an important role in permeability due to the strong plasticizing effect of organic vapors onto the PHBV film [48]. Similar water barrier enhancements were observed by Díez-Pascual et al. [81] who tested the water vapor permeability and the water uptake of PHBV nanocomposites containing ZnO, concluding that both parameters dropped gradually with increasing ZnO concentration in comparison to the neat biopolymer. Castro-Mayorga et al. [72] observed that the presence of a small amount of AgNPs to PHBV3/PHBV18 films could reduce water vapor permeability, reaching values close to the neat PHBV3 film. Melendez-Rodriguez et al. [47] reported that WVP and LP were also enhanced for concentrations over 7.5 wt.% of MCM-41 + eugenol into PHBV films.

The oxygen barrier enhancement mechanism created in nanocomposites is mainly attributed to the increased pathway (tortuosity) of the non-interacting permeant molecules to pass through the films. This mechanism is associated with factors, such as the effect of the microstructure in terms of dispersion, distribution, and aspect ratio of the impermeable nanofiller, its hygroscopic nature of it, the crystallization behavior of the polymer matrix, and the interfacial interaction across the nanofiller–polymer matrix [82]. The oxygen permeability (OP) of the pure PHBV and selected PHBV films containing CeO_2_NPs are found in Table 5. The neat PHBV film presented an OP value of 3.65 × 10^−19^ m^3^·m·m^−2^·Pa^−1^·s^−1^. After incorporation of 1.5 wt.% and 5.0 wt.% of CeO_2_NPs, the OP values increased to 6.92 × 10^−19^ m^3^·m·m^−2^·Pa^−1^·s^−1^ and 8.35 × 10^−19^ m^3^·m·m^−2^·Pa^−1^·s^−1^, respectively. The lower OP value in the film containing 1.5 wt.% CeO_2_NPs could be attributed to the better dispersion observed at low nanoparticle loading. In any case, the DSC results show that the enthalpy of fusion related to crystallinity decreased for the concentrations of nanofiller selected. As it is well-known, diffusion of non-interacting gas molecules occurs through the free volume in the amorphous phase of a semi-crystalline polymer matrix [83]. It is also known that the higher the nanofiller dispersion, distribution, and aspect ratio, the better the tortuosity factor is [84]. Thus, the interpretation could be that since the crystallinity of the nanocomposites is slightly lower, the oxygen molecule is very small and non-interactive, and some agglomeration was seen and expected at higher filler contents, the presence of the water-soluble cationic surfactant CTAB and the fact that the nanofiller has a square morphology, and no platelets could explain the somewhat higher oxygen permeability for the nanocomposites. Xu et al. [82] evaluated the oxygen barrier of platelets based on PHA and 5 wt.% GO-g-LAQ at 23 °C and 63.5% RH, obtaining an OP enhancement. However, in the case of PHA containing 5.0 wt.% GO-KH570 platelets, the OP was increased. Öner et al. [85] measured the OP at 23 °C and 0% HR of composites based on PHBV and boron nitride (BN) processed by melt blending. The oxygen permeability was reduced by 26.4% with 1 wt.% BN and 36.4% with 2 wt.% of BN, but it did not improve with a further increase in BN content, e.g., 3 wt.% BN. Similar observations were reported by Castro-Mayorga et al. [73] who concluded that the addition of 0.05 wt.% CuONPs to PHBV reduced the OP by 34.2% measured at 23 °C and 80% RH. However, an increase in the CuONPs concentration did not enhance the oxygen barrier properties of the PHBV. Considering the abovementioned studies, it becomes clear that the OP is extremely dependent on nanofiller concentration. Thus, a higher permeability was seen with an increase in the concentration, an effect often associated with nanofiller agglomeration and the formation of preferential paths for gas diffusion. The films developed here presented an intermediate oxygen barrier according to the ASTM D3985-05 standard [3]. In any case, the main aim for the use of this additive in this study was to exploit its active oxygen scavenging properties, for which higher concentrations demand to be used. Thus, from the overall results, it appears that a balance between active and passive oxygen barrier properties has to be accepted when using the material to design the most adequate food packaging.

### 3.9. Antimicrobial Activity of the Electrospun Films

Table 6 highlights the results of the antimicrobial activity of selected PHBV films containing CeO_2_NPs against *S. aureus* and *E. coli* strains. It can be observed that the PHBV films containing 1.5 wt.% CeO_2_NPs presented a significant reduction (R ≥ 1 and <3) of *S. aureus* and a slight reduction (R ≥ 0.5 and <1) of *E. coli*, whereas in PHBV films with 5.0 wt.% CeO_2_NPs, the reduction was significant (R ≥ 1 and <3) for both bacteria. The antimicrobial proficiency of CeO_2_NPs generally depends on their chemical and physical properties, i.e., specific surface area, size, morphology, and polar surface [86]. CeO_2_NPs can cause irreversible damage to bacteria membranes by different mechanisms of action, such as membrane dysfunction, nanoparticles penetration, interruption, blockage of transmembrane electron transport, ion release, and reactive oxygen species (ROS), such as the superoxide anion radical (O^2−^˙) and the hydroxyl radical (OH˙) [86,87,88]. The *S. aureus* showed slightly higher R values than *E. coli* because the cell wall morphology of *E. coli* is mainly formed of lipopolysaccharides and peptidoglycans that obstruct the diffusion of negatively charged reactive oxygen species created by the CeO_2_NPs [41]. Kızılkonca et al. [89] developed antibacterial films with CeO_2_NPs, chitosan, hydroxyethyl cellulose, and polyethylene glycol that reduced the growth of *E. coli* and *S. aureus* around 1.2 and 1.4 CFU/mL, respectively, after 12 h exposure. The films developed here containing CeO_2_NPs can also be used as packaging materials to avoid the growth of microorganisms.

### 3.10. Antioxidant Assay of the Electrospun Fibers and Films

**Figure 14:**
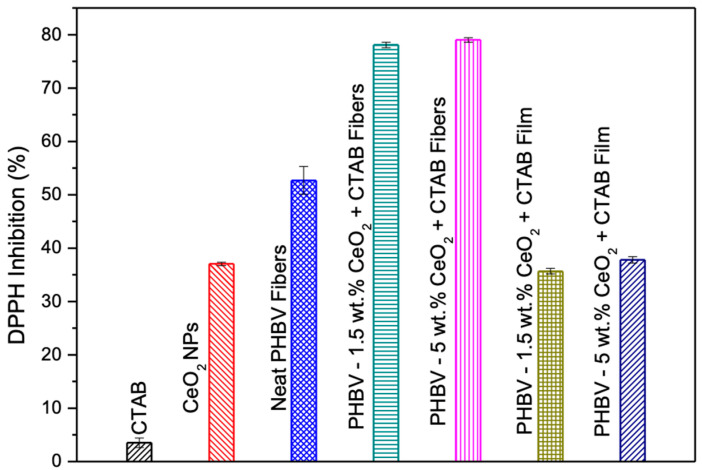
Comparison of the 2,2-diphenyl-1-picrylhydrazyl radical (DPPH) inhibition percentage (%) for hexadecyltrimethylammonium bromide (CTAB), cerium oxide nanoparticles (CeO_2_NPs), and electrospun poly(3-hydroxybutyrate-co-3-hydroxyvalerate) (PHBV) fibers and films containing 1.5 and 5.0 wt.% CeO_2_NPs.

The antioxidant activity of the CeO_2_NPs, CTAB, fibers, and selected films was determined by the DPPH free radical method. Figure 14 shows the percent inhibition of the free radical DPPH of the CTAB, CeO_2_NPs, neat PHBV fibers, and PHBV loaded with 1.5 wt.% and 5.0 wt.% CeO_2_NPs fibers and their corresponding annealed films. The CTAB did not show DPPH inhibition (~3.52%), while the CeO_2_NPs presented a DPPH inhibition of ~38% and the neat PHBV fibers of ~37.03%. The antioxidant activity increased in the electrospun PHBV fibers loaded with 1.5 wt.% and 5.0 wt.% CeO_2_NPs. In both cases, an increase in the antioxidant activity between 20% and 25% was observed in comparison to the neat PHBV fibers. When the films were formed, the antioxidant activity decreased, obtaining a DPPH inhibition of ~36.67% and 37.79% for films loaded with 1.5 wt.% and 5.0 wt.% CeO_2_NPs, respectively. Salevic et al. measured the antioxidant activity of PCL electrospun films containing sage extract, an essential oil with antioxidant activity. They also observed that the pure polymer did not possess antioxidant activity, which increased by increasing the content of essential oil until 80% [50].

Naidi et al. [86] measured the antioxidant activity of CeO_2_NPs by DPPH. A higher antioxidant activity was obtained with the increase of CeO_2_NPs, and the maximum scavenging activity of 55% was detected for 10 mg. Mohamed et al. [90] determined the total antioxidant capacity of CeO_2_NPs. The total antioxidant capacity was dependent on the nanoparticles concentration. The highest DPPH free-radical scavenging activity of ca. 36.07% was achieved at 400 μg mL^−1^. The selected films presented what could be considered a relevant antioxidant performance, which added to their oxygen scavenging and antibacterial properties, making them of interest for packaging applications, especially for extending shelf-life of products [91].

## 4. Conclusions

PHBV fibers loaded with different quantities of CeO_2_NPs were developed by electrospinning. The electrospinning technique allowed us to ensure the homogeneous distribution of the nanoparticles within the fibers. Additionally, this technique can generate ultrathin interlayers or coatings with bioadhesive properties, increasing the biobased content in the formulation and/or reducing the amount of raw materials required and potentially the cost. The electrospun fibers were transformed to biopapers of ~85 μm by means of an annealing treatment. The obtained PHBV films showed a uniform and continuous surface due to a thermally induced interfiber coalescence below the melting point and the degradation temperature of the biopolymer. The films showed contact transparency and a slight yellow color when loaded with the CeO_2_NPs. The thermal stability profile of the generated films was somewhat reduced by CeO_2_NPs, but all the PHBV films remained stable beyond 200 °C. The best morphological, barrier, mechanical, antimicrobial, antioxidant, and oxygen scavenging performance was attained for PHBV films containing 1.5 wt.% and 5 wt.% CeO_2_NPs + CTAB, which decreased the water vapor permeability but increased the limonene and oxygen permeability slightly. These films showed significant inhibition up to 15 days of evaluation against foodborne bacteria and a DPPH inhibition of over ~30%. The films became better oxygen by adding CTAB, achieving a significant headspace oxygen volume depletion even in film form. This is a preliminary work exploring the capacities of novel biopolymeric nanocomposites. Despite the raw materials being expensive at the lab scale, the active properties of the obtained materials could compensate for the potential higher cost. Therefore, the obtained electrospun biopapers could be used as a coating or an interlayer system for organic recyclable active packaging applications, which could extend shelf life and maintain the quality and safety of oxygen sensitive food items, such as such as chilled meat, hard cheese, dry mixes, coffee, snacks products, and fresh products, such as pasta and other food products that are packaged in for instance vacuum packaging and bag-in-box applications. In future works, the biodegradability of the full multilayer packaging concepts and the nanoparticles migration will be studied.

## Figures and Tables

**Table 1:** Optical characterization expressed in terms of the color parameters (a*, b*, L*, and ΔE*) and transparency results of electrospun poly(3-hydroxybutyrate-co-3-hydroxyvalerate) (PHBV) films loaded with cerium oxide nanoparticles (CeO_2_NPs) and hexadecyltrimethylammonium bromide (CTAB).

Sample	a*	b*	L*	ΔE*	T	O
Neat PHBV	0.74 ± 0.11	0.41 ± 0.15	90.44 ± 0.10	---	2.69 ± 0.11	0.019 ± 0.120
PHBV- 0.5 wt.% CeO_2_NPs	1.44 ± 0.08	−0.24 ± 0.25	90.41 ± 0.11	0.72 ± 0.10	8.05 ± 0.08	0.053 ± 0.210
PHBV- 0.5 wt.% CeO_2_NPs + CTAB	1.43 ± 0.10	−0.29 ± 0.14	90.51 ± 0.12	0.71 ± 0.09	8.13 ± 0.06	0.059 ± 0.150
PHBV- 1.0 wt.% CeO_2_NPs	1.02 ± 0.09	1.58 ± 0.11	90.27 ± 0.09	2.02 ± 0.07	9.83 ± 0.11	0.071 ± 0.190
PHBV- 1.0 wt.% CeO_2_NPs + CTAB	1.00 ± 0.05	1.57 ± 0.19	90.55 ± 0.06	2.00 ± 0.06	10.80 ± 0.15	0.072 ± 0.070
PHBV- 1.5 wt.% CeO_2_NPs	0.42 ± 0.12	3.89 ± 0.07	89.74 ± 0.20	4.36 ± 0.11	12.20 ± 0.20	0.088 ± 0.450
PHBV- 1.5 wt.% CeO_2_NPs + CTAB	0.26 ± 0.11	3.80 ± 0.15	89.94 ± 0.15	4.27 ± 0.08	12.50 ± 0.16	0.091 ± 0.310
PHBV- 2.0 wt.% CeO_2_NPs	0.18 ± 0.10	4.20 ± 0.11	89.24 ± 0.22	4.79 ± 0.10	10.10 ± 0.09	0.096 ± 0.240
PHBV- 2.0 wt.% CeO_2_NPs + CTAB	0.23 ± 0.19	4.10 ± 0.10	89.60 ± 0.36	4.62 ± 0.05	10.00 ± 0.11	0.104 ± 0.160
PHBV- 5.0 wt.% CeO_2_NPs	0.47 ± 0.17	4.88 ± 0.28	88.53 ± 0.32	5.63 ± 0.07	18.10 ± 0.14	0.146 ± 0.250
PHBV- 5.0 wt.% CeO_2_NPs + CTAB	0.59 ± 0.16	4.76 ± 0.21	88.68 ± 0.29	5.46 ± 0.09	17.80 ± 0.18	0.142 ± 0.370
PHBV- 10.0 wt.% CeO_2_NPs	−0.32 ± 0.11	10.86 ± 0.31	87.08 ± 0.17	11.81 ± 0.11	19.20 ± 0.31	0.166 ± 0.110
PHBV- 10.0 wt.% CeO_2_NPs + CTAB	0.36 ± 0.08	9.36 ± 0.18	87.77 ± 0.09	10.13 ± 0.06	19.80 ± 0.17	0.175 ± 0.150

a*: red/green coordinates (+a red, −a green); b*: yellow/blue coordinates (+b yellow, −b blue); L*: luminosity (+L luminous, −L dark); ΔE*: color difference; T: transparency; O: opacity.

**Table 2:** Thermal characterization of electrospun poly(3-hydroxybutyrate-co-3-hydroxyvalerate) (PHBV) fibers containing cerium oxide nanoparticles (CeO_2_NPs) and hexadecyltrimethylammonium bromide (CTAB) in terms of crystallization temperature (T_c_), melting temperature (T_m_), and normalized enthalpy of melting (ΔH_m_).

Sample	First Heating		Cooling	Second Heating	
	T_m1_ (°C)	ΔH_m1_ (J/g)	T_c_ (°C)	T_m2_ (°C)	ΔH_m2_ (J/g)
PHBV commercial	169.5 ± 0.3	78.1 ± 0.5	117.4 ± 0.1	171.5 ± 0.3	83.8 ± 1.1
PHBV- 0.5 wt.% CeO_2_NPs	174.6 ± 0.4	73.5 ± 0.9	118.6 ± 0.2	171.4 ± 0.2	74.1 ± 0.3
PHBV- 1.0 wt.% CeO_2_NPs	175.2 ± 0.3	71.7 ± 0.8	119.1 ± 0.1	171.9 ± 0.3	70.9 ± 0.6
PHBV- 1.5 wt.% CeO_2_NPs	175.3 ± 0.5	72.3 ± 0.6	118.5 ± 0.3	171.2 ± 0.4	71.7 ± 0.5
PHBV- 2.0 wt.% CeO_2_NPs	174.3 ± 0.4	69.7 ± 1.0	117.5 ± 0.4	170.3 ± 0.6	77.4 ± 0.3
PHBV- 5.0 wt.% CeO_2_NPs	176.8 ± 0.1	67.9 ± 0.4	118.6 ± 0.3	172.7 ± 0.2	70.1 ± 0.6
PHBV- 10.0 wt.% CeO_2_NPs	174.8 ± 0.7	63.3 ± 0.2	119.6 ± 0.5	171.1 ± 0.3	65.1 ± 0.4
PHBV- 0.5 wt.% CeO_2_NPs + CTAB	174.5 ± 0.6	69.6 ± 0.6	117.3 ± 0.4	167.3 ± 0.2	66.5 ± 0.7
PHBV- 1.0 wt.% CeO_2_NPs + CTAB	174.4 ± 0.7	69.7 ± 0.5	116.7 ± 0.3	164.4 ± 0.6	74.0 ± 0.4
PHBV- 1.5 wt.% CeO_2_NPs + CTAB	175.6 ± 0.6	68.7 ± 0.3	118.5 ± 0.2	169.1 ± 0.5	68.9 ± 0.8
PHBV- 2.0 wt.% CeO_2_NPs + CTAB	174.3 ± 0.2	71.2 ± 0.7	116.2 ± 0.5	165.7 ± 0.7	77.9 ± 0.2
PHBV- 5.0 wt.% CeO_2_NPs + CTAB	174.9 ± 0.3	66.1 ± 0.9	116.5 ± 0.7	162.9 ± 0.4	69.1 ± 0.5
PHBV- 10.0 wt.% CeO_2_NPs + CTAB	174.6 ± 0.4	64.0 ± 0.7	116.1 ± 0.3	162.9 ± 0.2	67.3 ± 0.8

**Table 3:** Thermal characterization of CeO_2_NPs, and electrospun poly(3-hydroxybutyrate-co-3-hydroxyvalerate) (PHBV) fibers containing cerium oxide nanoparticles (CeO_2_NPs) and hexadecyltrimethylammonium bromide (CTAB) based on mass loss at 5% (T_5%_), degradation temperature (T_deg_), and residual mass at 700 °C.

Sample	T5% (°C)	Tdeg (°C)	Residual Mass (%)
Neat PHBV	266.49	278.70	1.13
CeO_2_NPs	---	---	96.04
PHBV- 0.5 wt.% CeO_2_NPs	241.46	259.80	1.03
PHBV- 0.5 wt.% CeO_2_NPs + CTAB	229.93	252.22	1.85
PHBV- 1.0 wt.% CeO_2_NPs	232.72	258.26	2.03
PHBV- 1.0 wt.% CeO_2_NPs + CTAB	225.22	247.86	2.48
PHBV- 1.5 wt.% CeO_2_NPs	234.16	258.60	2.38
PHBV- 1.5 wt.% CeO_2_NPs + CTAB	215.92	239.33	3.02
PHBV- 2.0 wt.% CeO_2_NPs	239.71	265.35	2.93
PHBV- 2.0 wt.% CeO_2_NPs + CTAB	230.55	258.24	2.25
PHBV- 5.0 wt.% CeO_2_NPs	240.64	265.29	5.54
PHBV- 5.0 wt.% CeO_2_NPs + CTAB	237.82	265.51	4.43
PHBV- 10.0 wt.% CeO_2_NPs	236.96	261.66	15.56
PHBV- 10.0 wt.% CeO_2_NPs + CTAB	225.73	251.34	17.22

**Table 4:** Mechanical characterization of the electrospun poly(3-hydroxybutyrate-co-3-hydroxyvalerate) (PHBV) films loaded with cerium oxide nanoparticles (CeO_2_NPs) and hexadecyltrimethylammonium bromide (CTAB) based on tensile modulus (E), tensile strength at yield (σ_y_), elongation at break (ε_b_), and toughness (T).

Sample	E (MPa)	σ_y_ (MPa)	ε_b_ (%)	T (mJ/m^3^)
Neat PHBV	2394 ± 506 a	14.1 ± 4.0 a	1.01 ± 0.11 a	0.09 ± 0.04 a
PHBV- 1.5 wt.% CeO_2_NPs + CTAB	3309 ± 323 b	26.9 ± 4.5 b	1.22 ± 0.23 b	0.18 ± 0.08 b
PHBV- 5.0 wt.% CeO_2_NPs + CTAB	3546 ± 533 c	27.5 ± 3.7 c	1.19 ± 0.10 c	0.18 ± 0.03 b

a–c Different letters in the same column indicate a significant difference (*p* < 0.05).

**Table 5:** Comparison of the water vapor permeability (WVP), D-limonene permeability (LP), and oxygen permeability (OP) of the prepared samples based on electrospun poly(3-hydroxybutyrate-co-3-hydroxyvalerate) (PHBV) films loaded with cerium oxide nanoparticles (CeO_2_NPs) and hexadecyltrimethylammonium bromide (CTAB).

Sample	Thickness (µm)	WVP × 10^14^ (kg·m·m^−2^·Pa^−1^·s^−1^)	LP × 10^15^ (kg·m·m^−2·^Pa^−1^·s^−1^)	OP × 10^19^ (m^3^·m·m^−2^·Pa^−1^·s^−1^)
Neat PHBV	75	5.34 ± 1.79 a	26.80 ± 1.82 a	3.65 ± 0.51 a
PHBV- 1.5 wt.% CeO_2_NPs + CTAB	80	1.58 ± 0.54 b	6.71 ± 0.70 b	6.92 ± 0.36 b
PHBV- 5 wt.% CeO_2_NPs + CTAB	85	2.68 ± 0.83 c	8.23 ± 0.33 c	8.35 ± 0.23 c

a–c Different letters in the same column show a significant difference (*p* < 0.05).

**Table 6:** Antimicrobial properties of electrospun poly(3-hydroxybutyrate-co-3-hydroxyvalerate) (PHBV) films loaded with cerium oxide nanoparticles (CeO_2_NPs) and hexadecyltrimethylammonium bromide (CTAB) at 24 h exposure against *S. aureus* and *E. coli*.

Bacteria	CeO_2_NPs Content (wt.%)	Control Log (CFU/mL)	Film Log (CFU/mL)	R
*S. aureus*	1.5	7.94 ± 0.09	6.89 ± 0.11	1.05
	5.0		6.73 ± 0.07	1.11
*E. coli*	1.5	8.02 ± 0.10	7.09 ± 0.08	0.93
	5.0		6.98 ± 0.09	1.04

## Data Availability

Not applicable.
